# Antibody-nanoparticle conjugates to enhance the sensitivity of ELISA-based detection methods

**DOI:** 10.1371/journal.pone.0177592

**Published:** 2017-05-11

**Authors:** Margaret M. Billingsley, Rachel S. Riley, Emily S. Day

**Affiliations:** 1 Department of Biomedical Engineering, University of Delaware, Newark, Delaware, United States of America; 2 Department of Materials Science & Engineering, University of Delaware, Newark, Delaware, United States of America; 3 Helen F. Graham Cancer Center & Research Institute, Newark, Delaware, United States of America; Brandeis University, UNITED STATES

## Abstract

Accurate antigen detection is imperative for clinicians to diagnose disease, assess treatment success, and predict patient prognosis. The most common technique used for the detection of disease-associated biomarkers is the enzyme linked immunosorbent assay (ELISA). In an ELISA, primary antibodies are incubated with biological samples containing the biomarker of interest. Then, detectible secondary antibodies conjugated with horseradish peroxidase (HRP) bind the primary antibodies. Upon addition of a color-changing substrate, the samples provide a colorimetric signal that directly correlates to the targeted biomarker concentration. While ELISAs are effective for analyzing samples with high biomarker content, they lack the sensitivity required to analyze samples with low antigen levels. We hypothesized that the sensitivity of ELISAs could be enhanced by replacing freely delivered primary antibodies with antibody-nanoparticle conjugates that provide excess binding sites for detectible secondary antibodies, ultimately leading to increased signal. Here, we investigated the use of nanoshells (NS) decorated with antibodies specific to epidermal growth factor receptor (EGFR) as a model system (EGFR-NS). We incubated one healthy and two breast cancer cell lines, each expressing different levels of EGFR, with EGFR-NS, untargeted NS, or unconjugated EGFR antibodies, as well as detectable secondary antibodies. We found that EGFR-NS consistently increased signal intensity relative to unconjugated EGFR antibodies, with a substantial 13-fold enhancement from cells expressing high levels of EGFR. Additionally, 40x more unconjugated antibodies were required to detect EGFR compared to those conjugated to NS. Our results demonstrate that antibody-nanoparticle conjugates lower the detection limit of traditional ELISAs and support further investigation of this strategy with other antibodies and nanoparticles. Owing to their enhanced sensitivity, we anticipate that nanoparticle-modified ELISAs can be used to detect low levels of biomarkers found in various diseases, such as cancers, tuberculosis, and rheumatoid arthritis, and may ultimately enable earlier diagnosis, better prognostication, and improved treatment monitoring.

## Introduction

**Fig 1:**
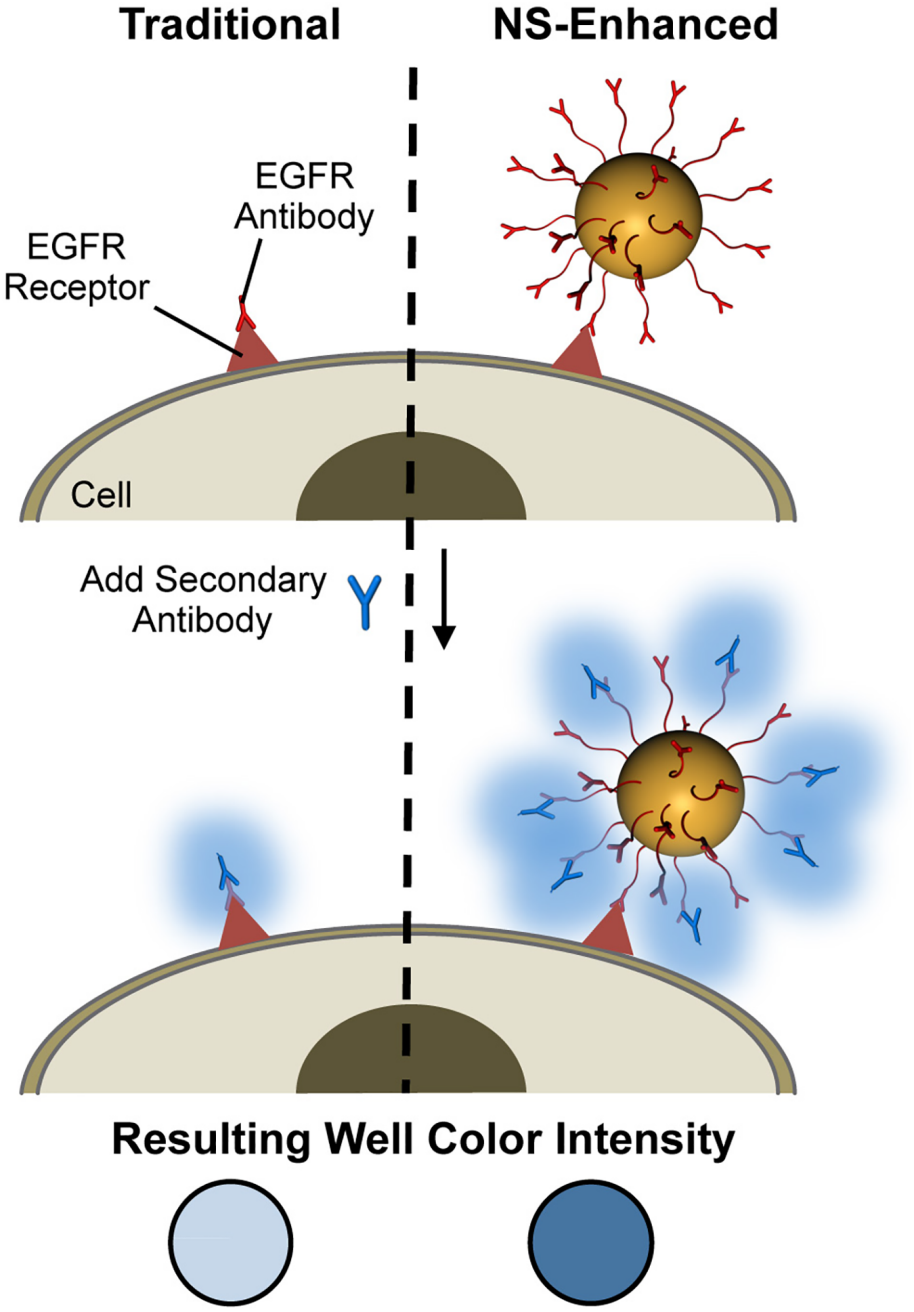
Schematic of the traditional and NS-enhanced ELISAs. In the traditional ELISA (left), freely delivered primary antibodies bind their target cell surface receptor and are then colorimetrically detected following the addition of secondary antibodies. Since there is a low ratio of detectible secondary antibodies per receptor, the resultant signal is low. In the NS-modified ELISA (right), NS are coated with primary antibodies to provide an abundance of binding sites for the detectible secondary antibodies, thereby enhancing the resultant colorimetric signal.

Antigen detection techniques are instrumental in biology and medicine to diagnose diseases, evaluate disease severity, and even predict patient outcomes. For example, clinicians currently use biomarker detection to diagnose and monitor diseases such as tuberculosis, rheumatoid arthritis, and metastatic cancers [1–5]. Additionally, biomarker detection techniques are used in non-disease applications such as pregnancy and blood tests [6,7]. The most simple and common antigen detection technique is the enzyme linked immunosorbent assay (ELISA) [2,5]. In a traditional ELISA, primary antibodies specific to the antigen of interest are combined with biological samples. Then, detectible secondary antibodies are added, which directly bind the primary antibodies. Finally, a color-changing substrate is added to generate a colorimetric signal, the intensity of which directly correlates with the level of target protein expression (Fig 1, left). The samples used in ELISAs often consist of immobilized antigens on the surface of a plate, but for biomarker detection in disease applications it is critical to be able to detect antigens directly on cell membranes, as overexpressed cell surface receptors distinguish diseased cells from normal cells. Moreover, the level of expression, and the percentage of cells with amplified expression, can indicate disease severity. Although ELISAs are widely used both in research and in clinical settings, they frequently lack the sensitivity necessary to detect targeted antigens expressed at low levels on cells or to detect low numbers of cells that express the targeted antigen [4,6,8]. Due to this low sensitivity, minor variations in protein expression or experimental protocol can yield inconsistent ELISA results [6]. This sensitivity issue can be attributed to a low signal-to-antigen ratio because each targeted biomolecule on the cell surface must come in direct contact with both primary and secondary antibodies to generate signal above background levels [4]. To overcome this limitation, we developed a modified ELISA that uses nanoparticles (NPs) coated with primary antibodies rather than unconjugated primary antibodies. Here, we demonstrate that these antibody-NP conjugates improve the detection limit of the traditional ELISA by providing an excess of binding sites for detectible secondary antibodies, ultimately leading to significant increases in the generated signal (Fig 1, right) [4,6,9].

The modified ELISA presented here was designed based on previous work that demonstrated NPs coated with targeting ligands can enhance the sensitivity of antigen-based detection assays [2,4,5,9–12]. A high number of ligands, such as antibodies, can be conjugated to the surface of each NP due to their high surface area-to-volume ratio [9,13,14]. These ligand-coated NPs can either be immobilized on a surface as part of a detection device [4,7,9] or incorporated as a solution directly into the assay protocol to improve antigen detection [2,5,6,10,11,15]. Here, we add NPs in solution to cells fixed to a surface to demonstrate the use of NPs to directly detect adherent cells expressing the biomarker of interest.

ELISA-based detection methods that utilize NPs in solution can be broadly classified as either one- or two-step assays. One-step assays eliminate the need for secondary antibodies by labeling biomarkers with NP conjugates that can be directly detected either because the NPs are attached to fluorophores or HRP molecules or because the NP itself has unique optical properties [2,10,11,16,17]. For example, Kim *et al*. developed NPs containing a fluorescent dye within a silica shell to detect circulating tumor cells (CTCs) in ovarian cancer. In their system, the authors precisely controlled the amount of RITC loaded into the silica shell to overcome autofluorescence in blood and then surface-coated the NPs with mucin 1 cell surface-associated antibody to specifically detect CTCs [11]. Similarly, ligand-coated surface-enhanced Raman scattering (SERS) NPs have been used to directly detect low levels of biomarkers with high accuracy and minimal background [10]. While each of these approaches offer the simplicity of one-step detection, the use of detectible primary antibodies can be cost prohibitive and the synthesis of SERS NPs requires both Raman reporter molecules and targeting ligands, making bulk synthesis complex and expensive. Furthermore, SERS detection requires expensive equipment that may not be readily available in clinical settings.

Compared to one-step methods, two-step detection methods use detectible secondary antibodies to bind primary antibodies (or other targeting ligands), and can yield higher specificity with decreased background interference. The most commonly used secondary antibodies for detection are fluorescently labeled or conjugated to an HRP molecule. A benefit of utilizing HRP conjugates rather than fluorescence is that it can overcome the substantial background fluorescence in whole blood that leads to high false-negative and false-positive rates [11,15,16]. Here, we developed a two-step detection assay that utilizes HRP-conjugated secondary antibodies to enable high specificity with low background.

We use HRP-anti-IgG conjugates as the detection mechanism because they are inexpensive, highly stable, and enable sensitive and consistent colorimetric detection, making them ideal for research or clinical use [2,5,6,16]. Additionally, we use gold-based NPs because they are simple to synthesize and characterize, and they are highly stable so that they can be stored for long periods of time without aggregation. Further, gold surfaces enable gold-thiol bioconjugation chemistry for simple functionalization with any ligand of interest. In this work, we used 150 nm diameter nanoshells (NS) composed of 120 nm silica cores and 15 nm thick gold shells as the NP platform because they have a large available surface area for antibody conjugation and their synthesis is simple, repeatable, and cheap, making them excellent candidates for clinical applications [8,18,19].

The objective of our study was to demonstrate that NS coated with primary antibodies can enhance the sensitivity of traditional ELISAs by providing an excess of primary antibody binding sites for secondary antibody detection, which can ultimately amplify the resultant colorimetric signal relative to freely delivered antibodies (Fig 1). We coated NS with antibodies specific to epidermal growth factor receptor (EGFR) because EGFR is an important biomarker in many diseases [20–23]. The EGFR family receptors, the ErbBs, are implicated in several cell signaling pathways, such as the MAPK, Akt, and JNK pathways, that regulate cell growth and apoptosis [24–26]. The overexpression or deregulation of ErbBs alters intracellular signaling cascades that are important for the progression of chronic kidney diseases, interstitial lung diseases, and many cancers [21–28].

In this work, we investigated the ability for EGFR-NS to bind breast cancer cell lines expressing different levels of EGFR and provide enhanced colorimetric signal relative to freely delivered EGFR antibodies, thus improving upon ELISA-based detection technology. Our results confirm that EGFR-NS can enhance the sensitivity of the traditional ELISA by amplifying the intensity of the colorimetric signal to detect cells expressing different levels of EGFR. Specifically, we demonstrate a 13-fold signal improvement from cells treated with EGFR-NS relative to cells treated with freely delivered EGFR antibodies. Additionally, the EGFR-NS-modified ELISA can detect cells that express low levels of EGFR, whereas the traditional ELISA cannot. These results support the use of ligand-functionalized NPs to improve upon ELISA-based detection methods. Given the importance of sensitive biomarker detection in guiding clinical decisions, the ability to enhance commercially available ELISAs by simply using antibody-NP conjugates rather then freely delivered primary antibodies could profoundly improve patient care.

## Materials and methods

### NS synthesis and characterization

NS were synthesized according to Oldenberg, *et al*. [29]. First, 2–3 nm diameter colloidal gold was prepared as described by Duff *et al*. by combining tetrakis(hydroxymethyl)phosphonium chloride (THCP, Sigma, St. Louis, MO, USA), 29.7 mM chloroauric acid (HAuCl_4_, Sigma), and 1 M sodium hydroxide (NaOH), and allowing it to age [30]. This colloidal solution was then reacted with 120 nm silica spheres functionalized with 3-aminopropyltriethoxysilane (NanoComposix Co., San Diego, CA, USA) in the presence of NaCl for 2–3 days at room temperature. Lastly, additional gold was reduced onto the NP surfaces using formaldehyde as the catalyst. Resultant silica core/gold shell NS were characterized by UV-visible spectrophotometry (Cary 60, Agilent Technologies, Santa Clara, CA, USA) and scanning electron microscopy (SEM, Hitachi S4700 Field-Emission Scanning Electron Microscope, Tokyo, Japan).

### Preparation of EGFR antibody-NS conjugates

To functionalize NS, mouse-anti-human EGFR antibodies (EGFR D-8, Santa Cruz Biotechnologies, Santa Cruz, CA, USA) were combined with 2 kDa orthopyridyl disulfide-PEG-*N*-hydroxysuccinimide (OPSS-PEG-NHS, Creative PEGWorks, Chapel Hill, NC, USA) or 2 kDa OPSS-PEG-succinimidyl valerate (OPSS-PEG-SVA, Laysan Bio, Arab, AL, USA) at a molar ratio of 2 PEG:1 antibody and were reacted overnight at 4°C to form OPSS-PEG-EGFR. Then, the OPSS-PEG-EGFR conjugates were added to the NS solution at 1250 molecules/NS and reacted for 4 hr at 4°C to construct EGFR-NS. Next, 5 kDa methoxy-PEG-thiol (mPEG-SH, JenKem, Plano, TX, USA) was added to the NS solution to a final concentration of 1 mM and reacted overnight at 4°C. Control NS were coated only with mPEG-SH (PEG-NS). Both EGFR-NS and PEG-NS were purified by centrifugation (1500 g, 5 min) to form a pellet and remove excess antibodies and PEG molecules with the supernatant, suspended in water, and stored at 4°C.

### Quantification of antibodies bound to NS

The mean number of EGFR antibodies bound per NS was quantified using an assay we have previously described [13]. Briefly, 4.1x10^9^ NS/mL were combined with 100 μg/mL horseradish peroxidase-conjugated goat-anti-mouse IgG (HRP-AM, Santa Cruz Biotechnology) in 3% BSA (bovine serum albumin) in PBS (phosphate buffered saline) (3% PBSA) for 1 hour. The samples were then centrifuged and re-suspended in 3% PBSA thrice to remove unbound secondary antibodies, and the supernatant was collected for background subtraction. The samples were then reacted with 3,3 ′,5,5 ′-tetramethylbenzidine dihydrochloride (TMB, VWR, Radnor, PA, USA) for 15 min. The reaction was stopped with 2 M sulfuric acid and sample absorbance at 450 nm was compared to a standard curve of known HRP-AM concentration. The absorbance of each sample was measured with a Cary 60 UV-vis spectrophotometer.

### Cell culture

BT20, MDA-MB-231, and HUVEC cell lines were purchased from American Type Culture Collection (ATCC, Manassas, VA, USA). BT20 and MDA-MB-231 breast cancer cells were cultured in Eagle’s Minimum Essential Medium (EMEM, Lonza, South Plainfield, NJ, USA) or Dulbecco’s Modified Eagle Medium (DMEM) respectively, and each media was supplemented with 10% fetal bovine serum (FBS) and 1% penicillin-streptomycin. Non-cancerous HUVECs were maintained in Endothelial Cell Growth Medium supplemented with 2% FBS, 0.04% hydrocortisone, 0.4% human fibroblastic growth factor-basic (FGF-b), 0.1% vascular endothelial growth factor (VEGF), 0.1% recombinant analog of insulin-like growth factor-1 (R3-IGF-1), 0.1% ascorbic acid, 0.1% human epidermal growth factor (hEGF), 0.1% Gentamicin, 15 μg/ml Amphotericin (GA-1000), and 0.1% heparin (Lonza) per manufacturer recommendations. Cells were maintained in a humidified incubator at 37°C with 5% CO_2_.

### Immunohistochemical staining

Immunohistochemical (IHC) staining was used to assess the inherent EGFR expression of each cell line. BT20s, MDA-MB-231s, or HUVECs were plated at 50,000 cells/well in a 24-well plate, incubated overnight in their appropriate culture medium, and fixed with 4% formaldehyde in ultrapure water. After fixation, cells were treated with 3% hydrogen peroxide for 10 min to block endogenous peroxidases, and then with 3% PBSA for 1 hr to block nonspecific antibody interactions. Cells were then exposed to 4 μg/mL EGFR antibodies diluted in PBSA for 1 hr at room temperature, rinsed 3 times with PBS, and treated with 1.5 μg/mL HRP-AM in 3% PBSA for 40 min. Lastly, 3-amino-9-ethylcarbazole (AEC, VWR) was added for 15 min to produce a red stain indicative of EGFR presence. Cells were imaged with a Zeiss Axioobserver Z1 Inverted Microscope equipped with a color camera.

### Western blotting

Western blotting was performed to corroborate the IHC results. BT20, MDA-MB-231, or HUVEC cells were lysed using lysis buffer (Amresco, Solon, OH, USA) supplemented with 0.1% protease inhibitor (Thermo Fisher Scientific, Waltham, MA, USA). Protein concentration was determined using a DC assay (Bio-Rad, Hercules, CA, USA) per manufacturer instructions. 15 μg of protein from each cell line was loaded into 8% Bis-Tris polyacrylamide gels (LifeTech, Carlsbad, CA, USA), and gels were run in 1X MES-SDS (2-(N-morpholino)ethanesulfonic acid-sodium dodecyl sulfate) running buffer for 35 min at 165 V. Protein was transferred onto nitrocellulose membranes for 15 min using a 1-step transfer system (Thermo Fisher Scientific). Membranes were blocked in 5% PBSA with 0.1% Tween-20 (PBST) for 1 hr and then incubated with 4 μg/mL goat-anti-mouse EGFR antibody (Santa Cruz Biotechnology) in 5% PBSA overnight at 4°C. After overnight incubation, membranes were washed 3 times with PBST and then incubated with 0.24 μg/mL HRP-AM (Santa Cruz Biotechnology) for 30 min at room temperature. Lastly, membranes were rinsed 3 times with PBST, reacted with VisiGlo electrochemiluminescent reagent (VWR) for 5 min, and imaged using the ChemiDoc-iT™2 Imager (UVP, Upland, CA, USA).

### Assessment of EGFR-NS binding to cells

To investigate the binding specificity of the EGFR-NS, each cultured cell line was fixed in 4% formaldehyde in ultrapure water, and then incubated with either no NS or with EGFR-NS or PEG-NS at 1.4 x 10^10^ NS/ml for 4 hours to allow binding of EGFR-NS to EGFR cell surface receptors. Next, we imaged the cells by light microscopy to visualize NS binding to cells. Although individual NS are extremely small (150 nm diameter), they are visible under light microscopy as black specs due to NP clustering upon binding cell surface receptors, which has been thoroughly described in previous literature [31]. Samples were imaged using a Zeiss Axioobserver Z1 microscope.

### Enzyme-linked immunosorbent assays

BT20, MDA-MB-231, or HUVEC cells were plated at either 5,000 or 20,000 cells per well in 96-well plates. After overnight incubation, the cells were fixed with 4% formaldehyde, blocked with 3% hydrogen peroxide for 10 min, and then blocked with 3% PBSA for 1 hr. In the traditional ELISAs, three wells each were treated with 8, 4, 2, or 1 μg/mL mouse-anti-human EGFR antibodies in 3% PBSA, corresponding to 25x, 50x, 100x, or 200x dilutions, respectively. For the NS-modified ELISAs, three wells each were treated with 1.4x10^10^ NS/mL EGFR-NS or PEG-NS, or free EGFR antibodies diluted 1000x. Since the antibody loading on NS was variable between batches, as described in the results, we adjusted the amount of free EGFR antibody added in each experiment from 0.2–0.5 μg/ml such that an equivalent amount of antibodies would be delivered as with the EGFR-NS. Cells were incubated with their respective treatment group for 1 hr at room temperature. Following at least 3 PBS washes, HRP-AM was added at 0.01 μg/mL for 1 hr at room temperature. Cells were washed again with PBS and then treated with TMB in the dark for either 40 min or 60 min for the traditional or NS-modified ELISAs, respectively. The samples’ absorbance was read on a Hybrid Synergy H1M plate reader at 650 nm. All experiments presented were repeated at least three times.

The data from the traditional ELISAs performed with unconjugated EGFR antibodies were analyzed by normalizing the raw absorbance data from each experiment to the absorbance measured in the highest antibody concentration. Then, the normalized data were averaged across three experiments. To analyze the NS-modified ELISA data, the background from cells treated without primary antibody was subtracted from the experimental samples (EGFR-NS, PEG-NS, or free EGFR) within each cell line. Then, we normalized the data to the HUVEC cells treated with free EGFR antibodies for each cell density so that we could show the resultant amplification beyond background levels. Finally, ANOVA with post-hoc Tukey-Kramer was performed in JMP software to reveal which experimental treatment groups in each cell line were statistically significantly different at the 95% confidence level.

## Results and discussion

### Characterization of EGFR-NS

**Fig 2:**
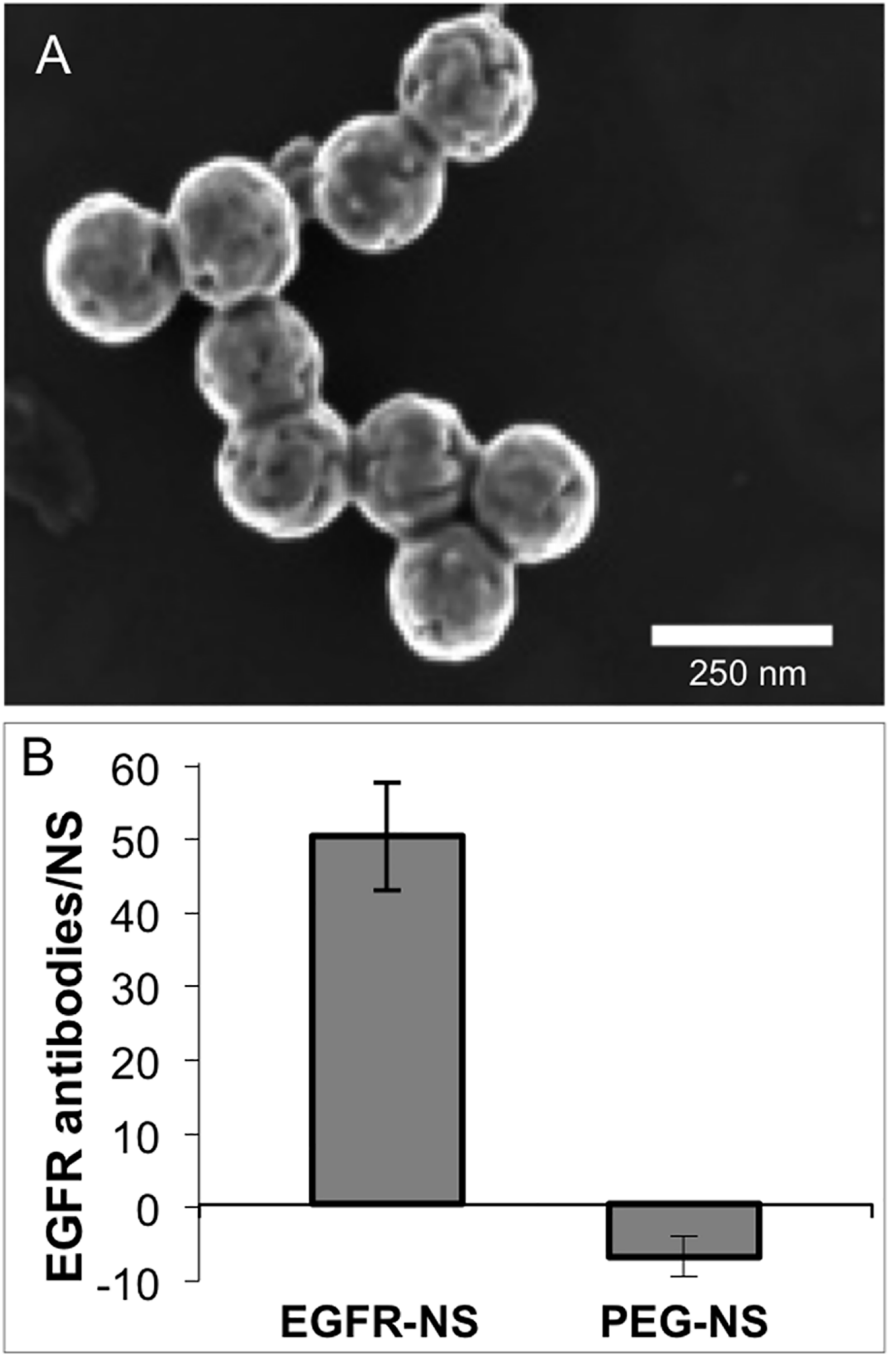
NS characterization. (A) Scanning electron micrograph of NS shows a homogenous size distribution and complete gold shells. (B) A representative solution-based ELISA shows that approximately 50 EGFR antibodies are bound to the surface of NS. PEG-NS showed minimal background.

Following synthesis, the NS were visualized by scanning electron microscopy (SEM, Fig 2A), which indicated they are ~150 nm in diameter with a homogenous size distribution. We qualitatively investigated antibody loading by UV-vis spectroscopy, which showed a red-shift in the peak NS absorbance following antibody conjugation (S1 Fig). Using the quantitative assay described in the methods, we determined that on average, the NS were coated with approximately 50 EGFR antibodies per NS (representative data is shown in Fig 2B). Since antibody loading was variable between batches of EGFR-NS, we varied the amount of free EGFR antibodies added to cells in each experiment so that the amount added was equivalent to the number delivered with EGFR-NS, as described in the Methods.

### EGFR-NS specifically bind cells expressing EGFR

**Fig 3:**
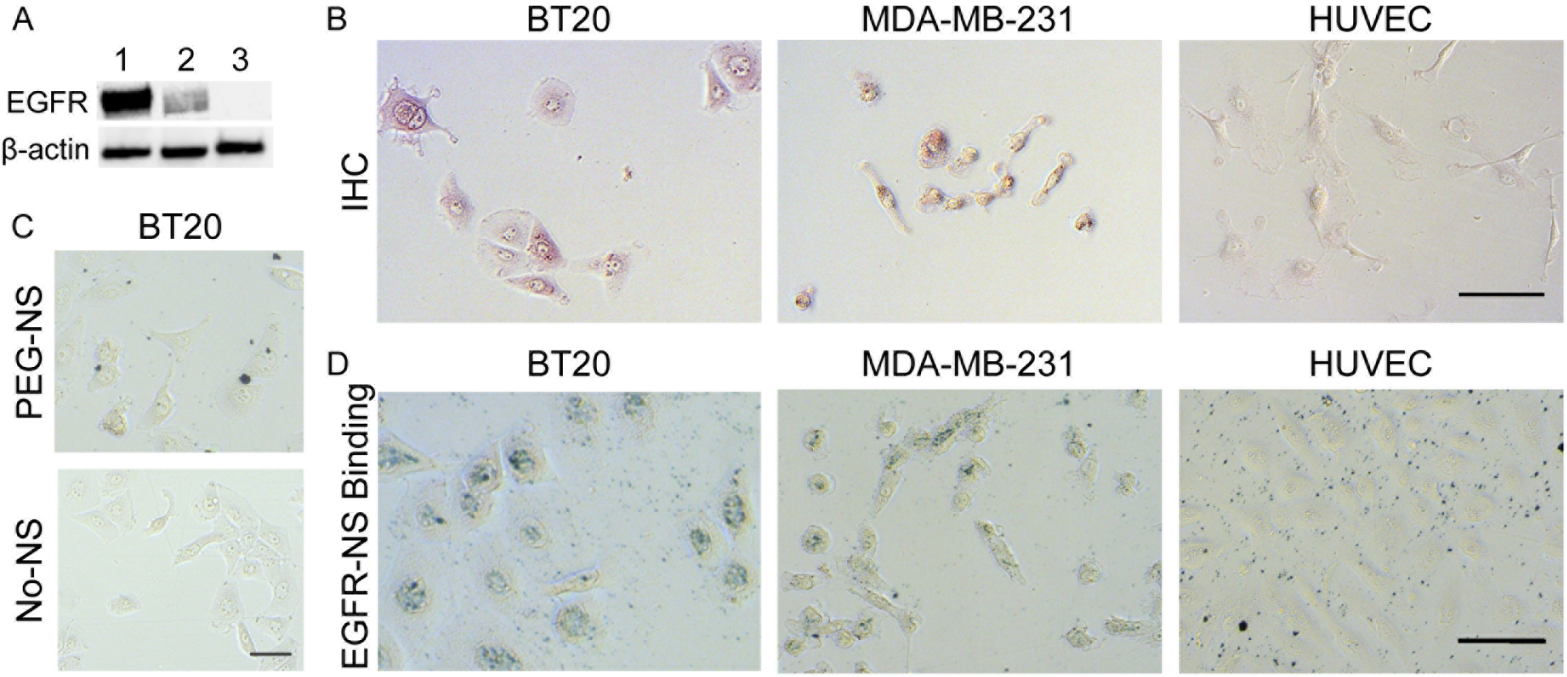
EGFR expression and EGFR-NS binding specificity. (A) Western blotting and (B) immunohistochemical staining show relative EGFR expression in BT20 (lane 1), MDA-MB-231 (lane 2), and HUVEC (lane 3) cell lines. Scale bar = 100 μm. In the Western blot, the top and bottom bands correspond to EGFR (170 kDa) and β-actin (42 kDa), respectively. (C) BT20 cells treated with non-targeting PEG-NS (top) and no NS (bottom). Scale bar = 50 μm. (D) Brightfield microscopy images showing specific binding of EGFR-NS (black specs) to each cell line. Scale bar = 100 μm.

To validate that EGFR-NS specifically bind cells expressing EGFR at levels corresponding to receptor expression, we analyzed EGFR expression and EGFR-NS binding in two breast cancer cell lines, BT20 and MDA-MB-231, and one non-cancerous cell line, human umbilical vein endothelial cells (HUVECs). We determined relative EGFR expression levels by Western blotting and immunohistochemical staining (Fig 3A and 3B), which both revealed that BT20 cells have high EGFR expression, MDA-MB-231 cells have moderate expression, and HUVECs have minimal expression, in agreement with literature (un-cropped and unedited Western blot provided as S2 Fig) [10,32]. This differential EGFR expression is critical to demonstrate the binding specificity of EGFR-NS and to correlate the level of binding with inherent EGFR expression. Additionally, since the accuracy of detection methods is often limited by high background signal, it was important to demonstrate that EGFR-NS amplify the resultant ELISA signal from cells expressing EGFR without increasing background noise from cells that do not express EGFR. Fig 3D and S3 Fig (which shows magnified sections of the images in Fig 3D) show that EGFR-NS (black specs) can bind BT20 cells, MDA-MB-231 cells, and HUVECs at levels corresponding to their EGFR expression (high, medium, low). Note that in the HUVEC samples, the EGFR-NS do not appear to be directly adhered to the cells, indicating that any resultant signal from these wells is background from EGFR-NS sticking to the well plate. Importantly, only EGFR-NS (Fig 3D), and not untargeted PEG-NS (Fig 3C), can bind BT20 cells, indicating that binding of these targeted NPs is primarily mediated by EGFR.

### EGFR-NS amplify ELISA signal compared to free EGFR antibodies

**Fig 4:**
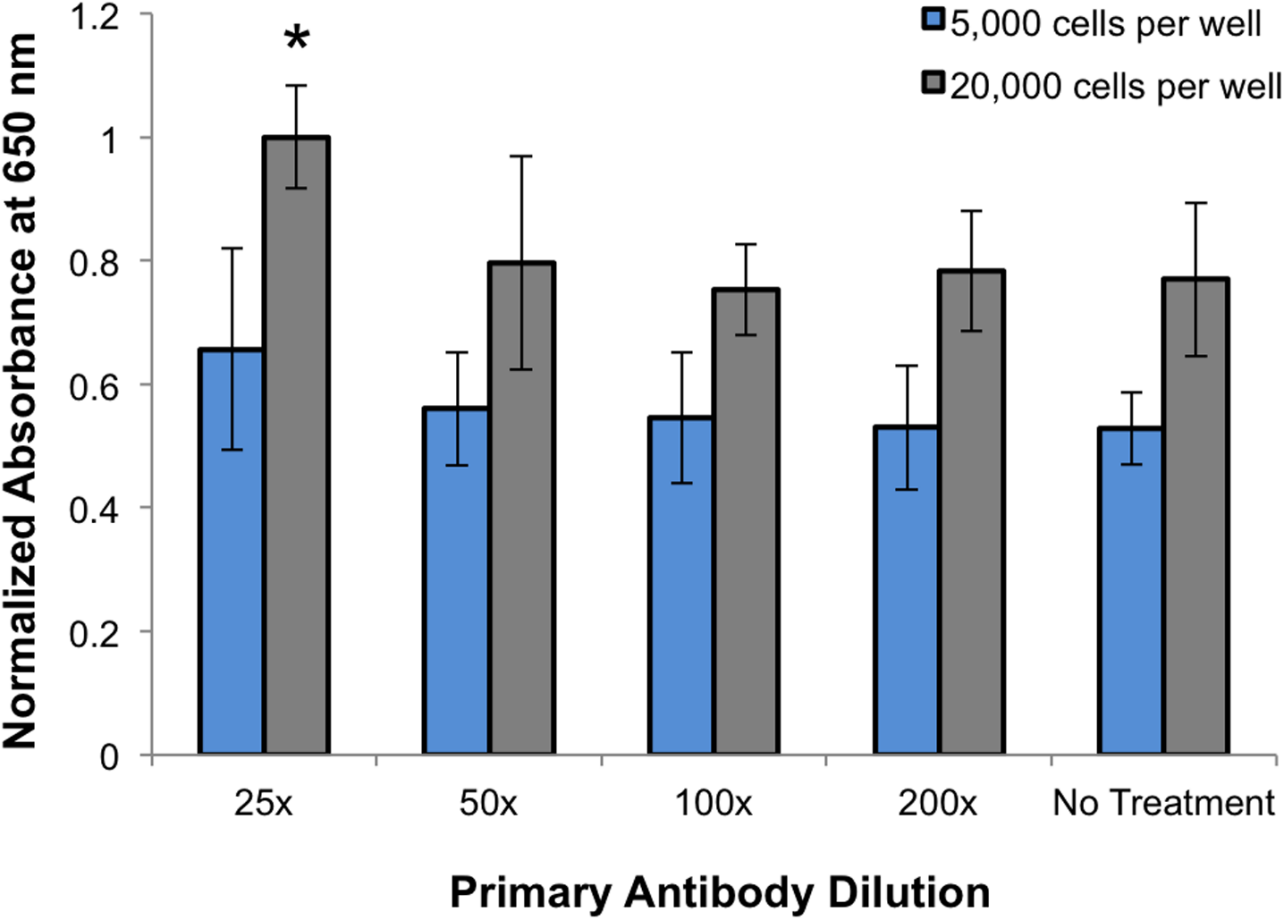
ELISA results from BT20 cells treated with 0, 1, 2, 4, or 8 μg/mL EGFR antibodies (corresponding to 25x, 50x, 100x, or 200x diluted) and 400x diluted HRP-conjugated secondary antibodies. The data shown are normalized to the highest EGFR antibody concentration. *p = 0.054 by t-test relative to untreated cells at the same density.

Our overarching hypothesis was that EGFR-NS could enhance the resultant signal relative to ELISAs conducted with unconjugated EGFR antibodies. Accordingly, ELISAs performed with EGFR-NS should detect fewer cells than those performed with unconjugated primary antibodies, even at lower antibody concentrations. To test this hypothesis, we first investigated the ability of a traditional ELISA performed with unconjugated EGFR antibodies at concentrations ranging from 0–8 μg/ml to detect EGFR-expressing BT20 cells plated at either 5,000 or 20,000 cells/well in 96 well plates. We found that only the highest concentration of freely delivered EGFR antibodies (8 μg/ml; corresponding to 25x dilution) produced a signal above background levels (Fig 4, p = 0.054 versus untreated control by t-test). Moreover, only BT20 cells plated at 20,000 cells/well, and not 5,000 cells/well, were detectable with the traditional ELISA even at this high EGFR antibody concentration. As detection-based assays must be sensitive enough to detect the low numbers of cells that are present early in disease progression, it appears that traditional ELISAs may be insufficient. The high concentration of antibody required would be cost prohibitive, which may limit practical clinical application. Therefore, it is desirable to enhance the sensitivity of ELISA-based detection methods to lower costs, detect fewer cells, and increase accuracy.

**Fig 5:**
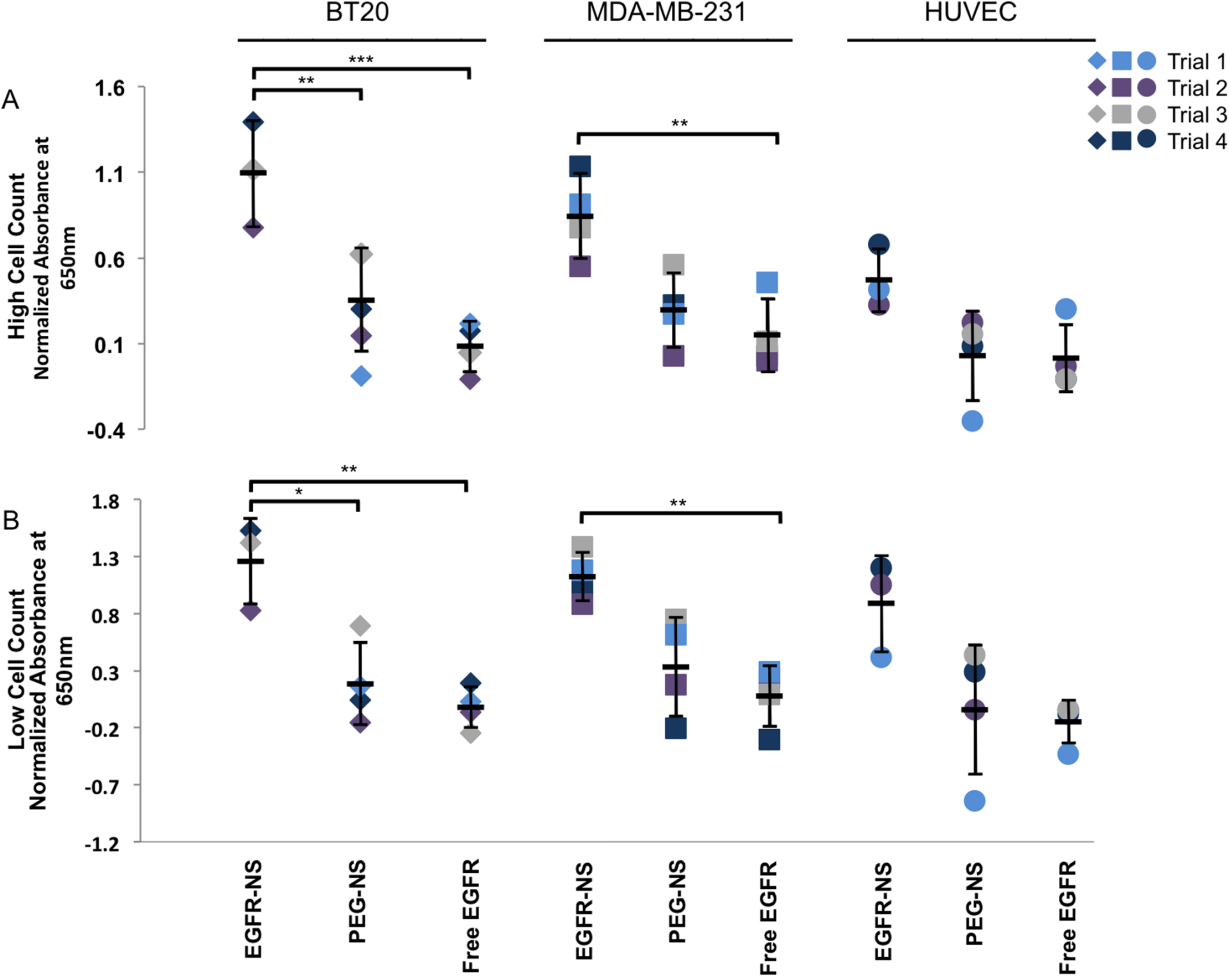
NS-modified ELISA compared to the traditional ELISA. Results from NS-modified ELISAs with (A) 20,000 cells/well and (B) 5,000 cells/well. Each cell line was treated with 1.4 x 10^10^ NS/ml of EGFR-NS or PEG-NS, or equivalent amount of free EGFR antibodies. Data are normalized to HUVEC cells treated with freely delivered antibodies, and each color represents individual experiments. *p<0.02, ^**^p<0.01, and ***p<0.001 by ANOVA with post-hoc Tukey-Kramer. Error bars represent standard deviations.

We conducted experiments, using EGFR-NS as a model system, to demonstrate that antibody-NP conjugates could improve the detection of cells with varying antigen expression relative to traditional ELISAs performed with unconjugated primary antibodies. BT20 cells, MDA-MB-231 cells, or HUVECs were treated with EGFR-NS, PEG-NS, free EGFR antibodies, or only PBS as described in the Methods. The amount of free EGFR antibody used (~1000x dilution) was equivalent to the amount of EGFR antibody present in wells treated with EGFR-NS. Interestingly, we found that ELISAs performed with unconjugated EGFR antibodies required 25x diluted antibodies to be effective (Fig 4), while ELISAs performed with EGFR-NS worked at 1000x antibody dilution (Fig 5). By incubating the same amount of unconjugated and NS-conjugated EGFR antibodies with the cells, we were able to directly compare the increase in resultant signal from the NS-modified ELISA to the traditional ELISA (Fig 5 (complete data from all experiments), S4 Fig (data presented as normalized means of all experiments), and S5 Fig (raw data from all experiments)). In both BT20 and MDA-MB-231 cells, the free EGFR antibodies failed to consistently produce a signal above the background level from HUVECs in samples plated at a density of 20,000 cells per well. Alternatively, EGFR-NS generated detectible signals in both cell lines, even when the number of plated cells was reduced to 5,000 per well. Since our overarching goal was to demonstrate the use of EGFR-NS to amplify the ELISA signal from cells overexpressing EGFR (such as BT20 and MDA-MB-231 cells), we were able to directly compare the resultant signal to the background signal from HUVECs treated with EGFR-NS, PEG-NS, or free EGFR, which would be reminiscent of nonspecific binding or NS sticking to the well plates. Notably, BT20 and MDA-MB-231 cells treated with EGFR-NS demonstrated a 13-fold (p = 0.0002) and 6-fold (p = 0.0075) increase in signal relative to freely delivered EGFR antibodies, respectively (Fig 5A, S4A Fig). Therefore, our results demonstrate that the level of signal amplification is directly correlated with EGFR expression. This concentration of unconjugated EGFR antibodies did not produce signal above background levels, demonstrating that EGFR-NS require ~40-fold fewer antibodies than a traditional ELISA.

Excitingly, we found similar trends in signal amplification for the lower cell density (5,000 cells/well, Fig 5B, S4B Fig). EGFR-NS significantly amplified the ELISA signal relative to free EGFR antibodies in BT20 (p = 0.002) and MDA-MB-231 (p = 0.0081) cells plated at this low density, although the amplification was not as pronounced as at the higher cell density. We expect that adding more EGFR-NS to the cells or increasing the number of antibodies loaded on the NS could further increase the signal amplification for low cell numbers. To ensure that the signal amplification observed in both the low and high cell density conditions was from the secondary antibodies binding the EGFR antibodies, and not from NS clustering, we completed the same treatment protocol without secondary antibodies. No difference in signal intensity was observed for cells treated with EGFR-NS and no secondary antibody, proving that the results from the NS-modified ELISAs may be attributed to the secondary antibodies specifically binding the primary antibodies conjugated to NS (S6 Fig). Overall, the results presented here demonstrate that EGFR-NS increase the sensitivity of ELISAs that typically utilize freely delivered antibodies. Further, EGFR-NS require much lower antibody concentrations to generate signal beyond background relative to freely delivered antibodies.

In this work, we developed EGFR-NS and investigated their ability to improve upon ELISA-based detection techniques to detect cells with varying EGFR expression levels. Notably, most one-step and two-step NP-based detection techniques reported to date have been limited to the detection of antigens immobilized on plates [2,5,6,12]. While immobilized antigens are useful for proof-of-concept detection studies, it is more clinically relevant to directly detect targeted antigens on diseased cells. Here, we present a two-step detection method that involves treating cells with primary antibody-conjugated NPs followed by HRP-conjugated anti-IgG secondary antibodies. NPs coated with primary antibodies inherently provide an excess of binding sites for secondary antibodies to adhere, and thereby amplify the signal obtained for each targeted cell surface receptor (Fig 1). We used EGFR as the targeted receptor because its’ overexpression is implicated in the progression of several diseases [20–23]. Therefore, EGFR has been studied as a biomarker for diagnosing and treating disease and is a promising target for detection-based assays [21,22,33]. We demonstrate that EGFR-NS can amplify the resultant signal relative to freely delivered antibodies to increase the sensitivity of ELISA-based detection methods.

In the future, this technique may be expanded to lower the detection limit of disease-associated biomarkers or cells that are present in complex fluids such as whole blood. For example, EGFR-NS could potentially be used to detect low levels of circulating tumor cells (CTCs), which indicate prognostic outcome in metastatic cancers, much earlier than they would be detectible with freely delivered EGFR antibodies [4,9,17,28,34–38]. Additional applications of ligand-conjugated NS and other NPs include assessment of rheumatoid arthritis, tuberculosis, respiratory syncytial virus, and any other diseases characterized by overexpressed biological markers [1–3]. Although we focused on EGFR in this work, we expect that NS coated with ligands can be used to enhance ELISA-based detection of any biomarker of interest simply by interchanging the targeting ligands conjugated to their surfaces. Additionally, the NP-enhanced ELISA we describe provides an inexpensive, simple, and accurate technique that can easily be adapted for clinical use.

## Conclusion

We demonstrate that NPs coated with antibodies can be used to lower the detection limit of ELISAs because they provide an excess of binding sites for detectible secondary antibodies. This amplifies the resultant signal and enables the differentiation of cells with varying receptor expression. We demonstrated this by performing an ELISA with three cell lines, each with differential EGFR expression, using either EGFR-NS or freely delivered EGFR antibodies. We observed that EGFR-NS could produce up to a 13-fold increase in signal intensity compared to unconjugated EGFR antibodies. Additionally, we found that ELISAs conducted with free EGFR antibodies require 40 times more antibody than present on EGFR-NS. Importantly, the ELISA signal generated with EGFR-NS correlated with the inherent EGFR expression of each cell line evaluated. Thus, the use of antibody-NP conjugates in ELISA-based applications would enable the detection of lower levels of targeted biomolecules compared to unconjugated antibodies while maintaining a high level of accuracy. Future studies will include investigating the use of EGFR-NS and other antibody-NP conjugates to detect diseased cells in complex solutions such as urine and whole blood to validate this technology in point-of-care detection. We anticipate that this technology can be used to improve upon ELISA technologies that are already used clinically to enable early detection of diseased cells, predict patient prognosis, and assess treatment efficacy.

## Supporting information

S1 Fig Nanoshells’ extinction red-shifts after antibody conjugation. (TIF)

S2 Fig Uncropped and unedited Western blot showing (A) EGFR bands at 170 kDa and (B) β-actin bands at 42 kDa in BT20 cells (lane 1), MDA-MB-231 cells (lane 2), and HUVECs (lane 3). After detecting EGFR, the same membrane was stripped with stripping buffer, rinsed 3X with TBST, and then stained for β-actin.
(TIF)

S3 Fig Magnified regions of the images shown in Fig 3D showing that EGFR-NS specifically bind BT20 and MDA-MB-231 cells, but only adhere to the well plate and not to cells in samples containing HUVECs, which have minimal EGFR expression. Scale bars = 100 μm.
(TIF)

S4 Fig Normalized means of NS-modified ELISA experiments. The results from each treatment group were averaged together and normalized to HUVEC cells treated with unconjugated antibody. The error bars represent the standard deviation in each treatment group. This graph provides another representation of the data presented in Fig 5.
(TIF)

S5 Fig Raw data from NS-modified ELISA experiments. The results from each trial are an average of the three wells of the biological replicate. This table provides the raw absorbance data (at 650 nm) presented in Fig 5 and S4 Fig.
(TIF)

S6 Fig BT20 cells plated at either 5,000 or 20,000 cells/well were treated with EGFR-NS, PEG-NS, free EGFR antibodies, or water, without secondary antibody treatment. The results shown are the raw absorbances at 650 nm following the addition of the color changing substrate.
(TIF)
