# Supplementary figures and images for: Antibody-nanoparticle conjugates to enhance the sensitivity of ELISA-based detection methods
Source: PLoS One. 2017 May 11;12(5):e0177592. doi: 10.1371/journal.pone.0177592 (PMC5426781; doi:10.1371/journal.pone.0177592)

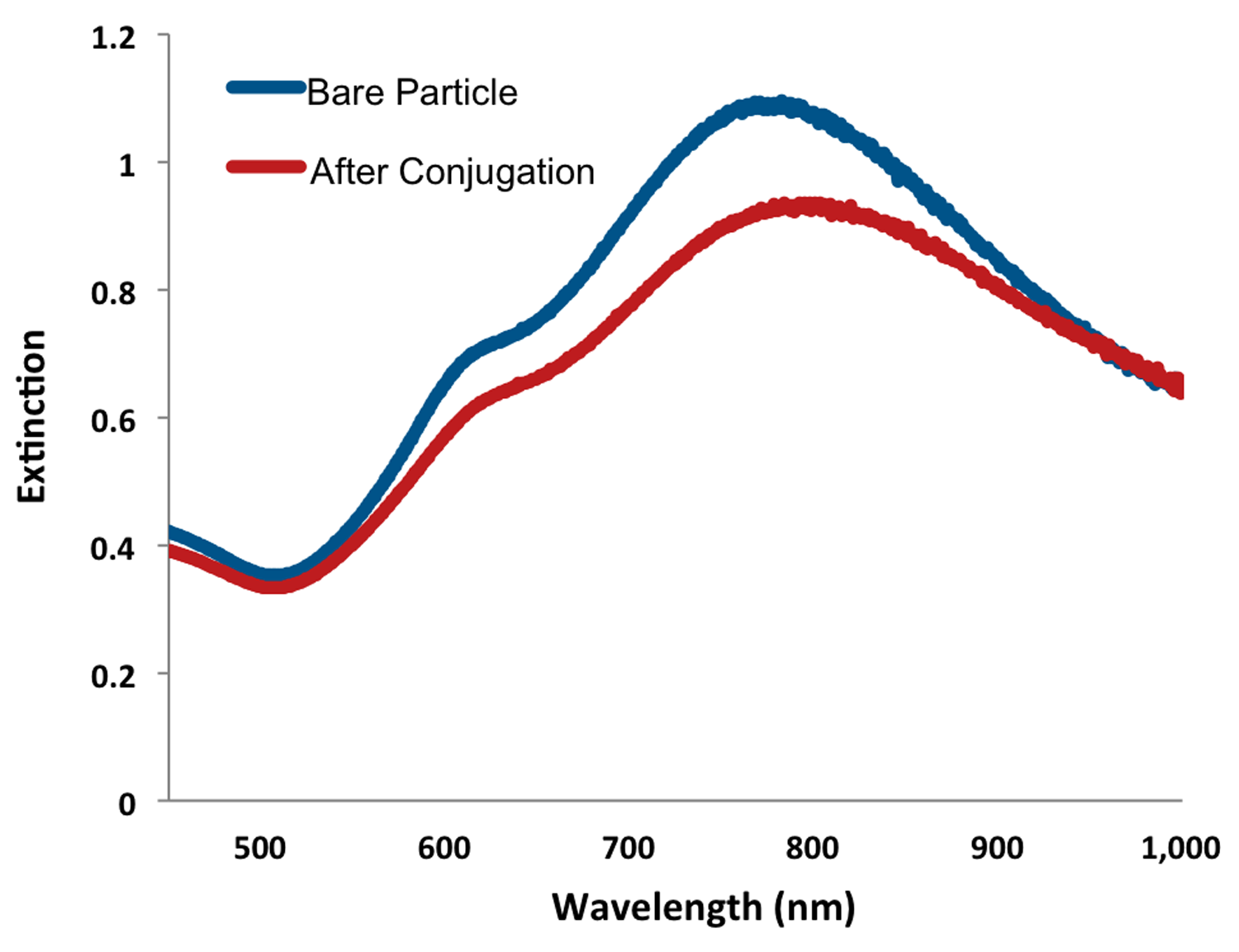

Supplement: S1 Fig — (TIF) [file pone.0177592.s001.tif]

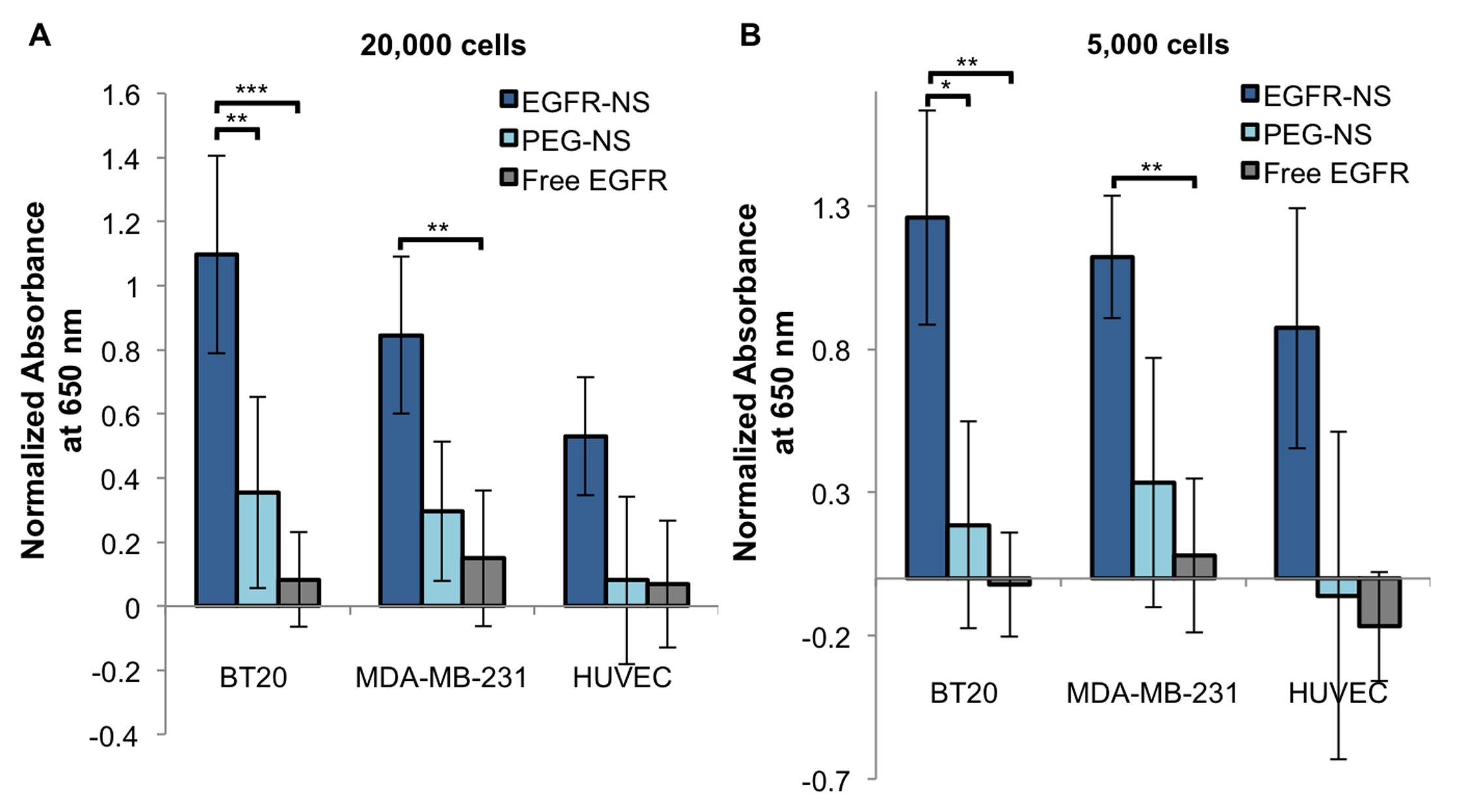

Supplement: S4 Fig — The results from each treatment group were averaged together and normalized to HUVEC cells treated with unconjugated antibody. The error bars represent the standard deviation in each treatment group. This graph provides another representation of the data presented in Fig 5. (TIF) [file pone.0177592.s004.tif]
